# Genome-wide identification and expression analysis of the *SR* gene family in longan (*Dimocarpus longan* Lour.)

**DOI:** 10.1371/journal.pone.0238032

**Published:** 2020-08-25

**Authors:** Xiaodong Chen, Shuqi Huang, Mengqi Jiang, Yukun Chen, Xu XuHan, Zihao Zhang, Yuling Lin, Zhongxiong Lai

**Affiliations:** 1 Institute of horticultural biotechnology, Fujian Agriculture and Forestry University, Fuzhou, China; 2 Institute de la Recherche Interdisciplinaire de Toulouse, Toulouse, France; Florida Atlantic University, UNITED STATES

## Abstract

Longan (*Dimocarpus longan* Lour.) is an important commercial fruit tree in southern China. The embryogenesis of longan affects the quality and yield of fruit. A large number of alternative splicing events occurs during somatic embryogenesis (SE), which is regulated by serine/arginine-rich (SR) proteins. However, the functions of SR proteins in longan are poorly understood. In this study, 21 *Dlo-SR* gene family members belonging to six subfamilies were identified, among which Dlo-RSZ20a, Dlo-SR30, Dlo-SR17, Dlo-SR53 and Dlo-SR32 were localized in the nucleus, Dlo-RSZ20b, Dlo-RSZ20c, Dlo-RSZ20d, Dlo-SC18, Dlo-RS2Z29, Dlo-SCL41, and Dlo-SR33 were localized in chloroplasts, and Dlo-RS43, Dlo-SC33, Dlo-SC37, Dlo-RS2Z33, Dlo-RS2Z16, Dlo-RS2Z24, Dlo-SCL43, Dlo-SR112, and Dlo-SR59 were localized in the nucleus and chloroplasts. The *Dlo-SR* genes exhibited differential expression patterns in different tissues of longan. The transcript levels of *Dlo-RSZ20a*, *Dlo-SC18*, *Dlo-RS2Z29*, *DLo-SR59*, *Dlo-SR53*, and *Dlo-SR17* were low in all analyzed tissues, whereas *Dlo-RS43*, *Dlo-RS2Z16*, *Dlo-RS2Z24*, and *Dlo-SR30* were highly expressed in all tissues. To clarify their function during SE, the transcript levels of *Dlo-SR* genes were analyzed at different four stages of SE, comprising non-embryonic callus (NEC), friable-embryogenic callus (EC), incomplete compact pro-embryogenic culture (ICpEC) and globular embryo (GE). Interestingly, the transcript levels of *Dlo-RS2Z29* and *Dlo-SR112* were increased in embryogenic cells compared with the NEC stage, whereas transcript levels of *Dlo-RSZ20a*, *Dlo-RS43*, *Dlo-SC37*, and *Dlo-RS2Z16* were especially increased at the GE stage compared with the other stages. Alternative splicing events of *Dlo-SR* mRNA precursors (pre-mRNAs) was detected during SE, with totals of 41, 29, 35, and 44 events detected during NEC, EC, ICpEC, and GE respectively. Protein–protein interaction analysis showed that SR proteins were capable of interaction with each other. The results indicate that the alternative splicing of *Dlo-SR* pre-mRNAs occurs during SE and that Dlo-SR proteins may interact to regulate embryogenesis of longan.

## 1 Introduction

Alternative splicing (AS) allows the production of a large number of protein isoforms from a small number of genes. Alternative splicing is the most important factor leading to the complexity of organisms, generating complex proteomes and functioning in quantitative gene control during differentiation, development, and disease [1]. Multiple proteins can be expressed from one gene through AS under different circumstances, thereby increasing the complexity and adaptability of the organism under diverse physiological conditions.

Alternative splicing is generally regulated by splicing factors, of which one category is highly conserved serine/arginine-rich (SR) proteins. These SR proteins play vital roles in spliceosome assembly and mRNA splicing, and influence the selection of AS sites through changes in concentration of SR proteins [2]. Serine/arginine-rich proteins are best characterized in mammals and are defined by the presence of RNA binding domains (RBDs) at the N-terminus and adjacent arginine and serine repeats (the RS domain) [3, 4]. On the basis of the characteristics of the RS domain and the functions of the other domains in mammals, SR proteins can be classfied into three categories. (1) Classical SR proteins contain one or two RBDs at the N-terminus and one RS domain at the C-terminus. Classical SR proteins can be recognized by the monoclonal antibody mb104 and participate in constitutive and alternative RNA splicing. The first identified classical SR protein to be identified was SF2/ASF [5]. (2) SR-like proteins contain one RS domain. However, the protein is either not recognized by the mb104 monoclonal antibody or is only involved in one of constitutive or alternative splicing. Additional types of RBD, such as the PWI domain, are present in these proteins [6]. (3) Other proteins contain one or more RS domains and additional domains that perform different functions, e.g., Prp5 contains an ATPase/helicase domain [2]. Similar to SR proteins in mammals, the conserved RBD domain exists in plant SR proteins. However, the RS domains in plants are more diverse and six subfamilies of SR proteins, comprising SR, RSZ, SC, SCL, RS2Z and RS, have been proposed in *Arabidopsis* based on domain analysis [7]. The SR and RS subfamilies contain proteins with two RBDs, whereas the former subfamily possesses a conserved motif (SWQDLKD) in the second RBD. The SC subfamily consists of SR proteins containing one RBD and one RS domain. Proteins of the RSZ, SCL, and RS2Z subfamilies contain a single RBD and a typical RS domain. The differences among the RSZ, SCL, and RS2Z subfamilies are that one zinc knuckle is present in RSZ proteins, a N-terminal charged extension is present in SCL proteins and two zinc knuckles are contained in RS2Z proteins [7].

The involvement of SR proteins in embryogenesis has been reported in metazoans previously. In *Caenorhabditis elegans*, RNA interference of *SF2/ASF* leads to lethal effects in late embryogenesis [8]. In mouse, *SRp20* was observed in egg cells and early embryos to exert a maternal cytoplasmic genetic effect. Depletion of SRp20 derived from the maternal line prevents normal embryo development and embryogenesis is arrested before the blastocyst stage. Attempts to use knockout techniques to breed *SRp20*-deficient mice failed [9]. Mice defective in *SRSF1*, *SRSF2*, *SRSF3*, or *SRSF10* exhibit an embryonic lethal phenotype, which suggests that these proteins are essential for embryonic development [9–11]. The cell cycle of the chicken DT40 cell line lacking *SRp38* is significantly changed with a prolonged G2/M phase [12]. To date, research on AS and SR proteins in plants has predominantly focused on stress response, the flowering mechanism and development in *Arabidopsis* [13–16]. The life cycle of woody plants differs from that of the annual *Arabidopsis* and must be responsive to environmental fluctations. However, knowledge of AS events and SR proteins of woody plants is scarce.

Longan (*Dimocarpus longan* Lour.) is an important commercial fruit tree in southern China. Although the fruit show high nutritional value and are beneficial for human health, problems such as variable inter-annual yields and low proportion of edible pulp seriously restrict expansion of the longan industry. Embryo development is strongly associated with the yield and fruit quality of longan. Plant somatic embryos show close morphological and molecular similarities to normal zygotic embryos [17–20]. Somatic embryos of longan have been used to investigate the embryogenesis mechanism *in vitro* and *in vivo* [21–23]. Through analysis of the longan transcriptome, a large number of AS events have been detected during somatic embryogenesis (SE), which can be divided into six stages including friable-embryogenic callus (EC), incomplete compact pro-embryogenic cultures (ICpEC), globular embryos (GE), heart-shaped embryos, torpedo-shaped embryos and cotyledonary embryos [24]. Among these six stages, the first three stages (EC, ICpEC and GE) belong to the early stage during SE and the cultivation of early stage embryo requires the addition of plant hormone 2,4-dichlorophenoxyacetic acid (2,4-D). In the present study, genome-wide identification and analysis of the *Dlo-SR* gene family members were performed and the expression pattern of *Dlo-SR* genes in different tissues of longan was analyzed. In addition, AS events of *Dlo-SR* genes were analyzed during SE in longan and the potential interactions among selected Dlo-SR proteins were predicted.

## 2 Materials and methods

### 2.1 Experimental materials

Friable-embryogenic callus of *D*.*longan* ‘Honghezi’ (LC2 cell line), induced by Lai Zhongxiong in 1994, was long-term sub-cultured by the Institute of Horticultural Biotechnology, Fujian Agriculture and Forestry University [21–22, 25]. In the present study, callus of the LC2 cell line was first cultured on Murashige and Skoog (MS) medium supplemented with 2,4-D (1.0 mg/L), kinetin (0.5 mg/L), and AgNO_3_ (5 mg/L) for 20 days and was subsequently transferred to MS medium supplemented with 2,4-D (1.0 mg/L) for an additional 20 days to obtain friable-embryogenic callus (EC). The EC was cultured on MS medium supplemented with 2, 4-D (0.5 mg/L) for an additional 20 days to obtain incomplete compact pro-embryogenic cultures (ICpEC). To obtain cells at the globular embryo (GE) stage, the EC was cultured on MS medium supplemented with 2,4-D (0.1 mg/L) for 20 days. Mature embryos were cultured on MS medium for about 45 days to obtain non-embryonic callus (NEC) [24–26]. The cultivation of synchronized cultures at NEC, EC, ICpEC, and GE stages were conducted for three replications. The samples for qRT-PCR analysis were generated from five bottles of synchronized culture, respectively as described in the previous study [24].

### 2.2 Identification of *Dlo-SR* gene family members

Longan genome data were downloaded from the GigaScience GigaDB repository (2017) (http://dx.doi.org/10.5524/100276). Longan SR family members were isolated from the genome data as follows. First, the known SR accession number of Arabidopsis and rice were used to search the amino acid sequences from TAIR (http://www.arabidopsis.org/, V10.0) and RGAP (http://www.rice.plantbiology.msu.edu), respectively [7]. Second, a longan genome-wide amino acid sequence database was generated using BLAST (Standalone). Alignment of the longan sequences with the amino acid sequences of known Arabidopsis and rice SR proteins (E-value = 0.001) confirmed the candidate longan SR family members. Third, the candidate Dlo-SR family members were verified using the Pfam database (PF00076.21). A total of 21 candidate sequences were obtained finally and the CDS sequences of *Dlo-SRs* were showed in S1 Table. Transcriptome data for nine tissues and organs of longan ‘Si Ji Mi’ were accessed in the National Center for Biotechnology Information (NCBI) GEO database (accession number GSE84467). The AS events of *Dlo-SR* genes at the NEC, EC, ICpEC, and GE stages were extracted from the longan transcriptome dataset (NCBI Accession No.: SRA050205).

A controlled plant chamber was used for cultivation of longan ECs under different light conditions. The intensity and photoperiod of blue (457 nm) and white light were fixed at 32 μmol m^−2^ s^−1^ and 12 h d^−1^, respectively. Transcriptome data under different light qualities (white, blue, and dark) were extracted from the NCBI BioProject database (accession number PRJNA562034).

### 2.3 Bioinformatics analysis

Protein physicochemical properties were predicted using the ExPASy Protparam online tool (https://web.expasy.org/protparam/). Subcellular localization was predicted with the Plant-mPLoc server (http://www.csbio.sjtu.edu.cn/bioinf/plant-multi/) [27–29]. Protein phosphorylation sites were predicted using the NetPhos2.0 server (http://www.cbs.dtu.dk/services/NetPhos-2.0/) [30]. The neighbor-joining method implemented in MEGA7.0 was used to construct a phylogenetic tree for the longan and *Arabidopsis* SR proteins from p-distances between all pairs of sequences. The reliability of the tree topology was evaluated by means of a bootstrap analysis with 1000 replicates. Exon and intron position information was mapped using the GSDS server (http://gsds.cbi.pku.edu.cn/) [31]. Conserved motifs were predicted using the MEME tool (http://meme-suite.org/tools/meme). The 2000 bp genomic sequence upstream of the start codon for each *SR* gene was extracted as the putative promoter region and the PlantCARE database (http://bioinformatics.psb.ugent.be/webtools/plantcare/html/) was used to predict the promoter *cis*-acting elements [32].

### 2.4 RNA extraction and gene expression analysis

Total RNA was extracted from cultures at the longan NEC, EC, ICpEC, and GE stages using TriPure Isolation Reagent (Roche Diagnostics, Indianapolis, IN, USA). Extracts were treated with DNase I to remove any contaminating genomic DNA. The RNA quality was analyzed using a Nanodrop 2000 spectrophotometer (Thermo Scientific, Wilmington, DE, USA). The cDNA was synthesized with the PrimeScript RT Reagent Kit (TaKaRa, Japan), using 500 ng RNA in a 10 μL reaction volume. Transcript levels were analyzed by qRT-PCR performed on a Lightcycler 480 system (Roche Applied Science, Basel, Switzerland). The 22.5 μL final reaction volume contained 12.5 μL SYBR II Premix Ex Taq™ (Takara), 1 μL of 10× diluted cDNA, 0.8 μL specific primer pairs (100 nM), and 7.4 μL ddH_2_O [33]. The qRT-PCR protocol was as follows: 95°C for 30 s, followed by 40 cycles of 95°C for 5 s, 60°C for 20 s and 72°C for 10 s. Each qRT-PCR analysis was performed in biological triplicates and technical replications. The *β-actin* (*ACTB*) gene was used as an internal control for calculation of the relative expression level of *SR* genes following the 2^−△△*C*t^ method [33]. The data presented are the average ± standard error of three replicates. The primers for qRT-PCR were designed using Primer 3 software (Table 1).

**Table 1:** Primers for detection of *SR* genes in longan.

Gene NO.	FP Sequence (5'to3')	RP Sequence (5'to3')
**Dlo_000762.1**	CTTCGACAAGTACGGCAAGG	GGTACTTGTGACGGTCAGGA
**Dlo_004147.1**	GTCGTGGAAGGAGTGAGACT	TCCACAGTGATATACCGGCC
**Dlo_006257.1**	GTCAAGTGATCGTCGTGGTG	CCTCACTGTCTCGGGAAACT
**Dlo_006259.1**	GTCAAGTGATCGTCGTGGTG	CCTCACTGTCTCGGGAAACT
**Dlo_006464.1-F**	TCAAGAAGCCCGAGTCGTAG	CCTTGAAACAGACCGTGGTG
**Dlo_007311.1-F**	CTGAGAGAGGCCGTGATAGG	TGTAAGGACTCTGGCTGGTG
**Dlo_007965.1-F**	TCCTGACCGTCACAAGTACC	AACCCTGGATCTATGCGGAG
**Dlo_008139.4-F**	TGACCACGGTTGCATTTGTT	GAGCGATCCCTATGCCTCTT
**Dlo_009794.2-F**	GAGGTGGAGGTGGAGGTTAC	AATTTCCCTCCCCGCAAAAC
**Dlo_010896.1-F**	TGGTCGGGATGTTGATGGAA	CATCTGGTGTTAGACTGCGC
**Dlo_020652.1-F**	GAGGGACAGAGACTATCGCC	CCGTTCATCATGACTGCGTC
**Dlo_021112.1-F**	GGTATTCTCCGAGGCGAAGA	GCCAAATCCACGAGAATCCC
**Dlo_021971.1-F**	AGATTCTCCTTACCGCCGAG	AAGGAAGGGGAGAACGTCTG
**Dlo_022256.1-F**	GTTGGTGGGTCTGATCAGGA	CCAAGCAAACCAGGAAACGA
**Dlo_029439.1-F**	CGCTATTTGGGGCTGAGAAC	TGCGCTCCTAGTCTCATACG
**Dlo_030358.1-F**	TATCTGTGGAGTGGGCAAGG	TGTTGCATCCTCCTGTGTCT
**Dlo_036683.1-F**	GTGTTATGAGTGTGGCGAGC	TACGACGAGGAGGAGGAGAT
**Dlo_032826.1-F**	GGTGGTCGTTTTGGTGTCTC	TCGACGGACTGCCATCATAT

### 2.5 Construction of interaction networks of SR proteins in longan

STRING (https://string-db.org/) was used to construct the functional protein association networks for SR proteins on the basis of *Arabidopsis *orthologs. The minimum required interaction score was set to Medium confidence (0.400) and the maximum number of interactors was 10.

### 2.6 Statistical analysis

Statistical analysis was performed using SPSS (version 19.0, Chicago, IL, USA). The gene expression data was analyzed by one-way analysis of variance (ANOVA) Different lower-case letters were used to indicate the differences significant at *P* < 0.05.

## 3 Results

### 3.1 Basic information of *Dlo-SR* gene family members

Twenty-one candidate sequences for *Dlo-SR* gene family members were confirmed. Analysis of the physicochemical properties of the Dlo-SRs protein with ExPASy Protparam revealed that the length of Dlo-SR proteins ranged from 141 amino acids (Aa) (Dlo-RS2Z16) to 1,013 Aa (Dlo-SR112), the molecular weight varied from 16.16 kDa (Dlo-RS2Z16) to 112.05 kDa (Dlo-SR112), and the isoelectric point ranged from 6.19 (Dlo-SR53) to 12.28 (Dlo-SCL43) (Table 2). According to the Plant-mPLoc prediction analysis, five family members (Dlo-SR30, Dlo-SR17, Dlo-SR53, Dlo-SR32, and Dlo-RSZ20a) are localized in the nucleus, seven members (Dlo-SR33, Dlo-SCL41, Dlo-RS2Z29, Dlo-SC18, Dlo-RSZ20b, Dlo-RSZ20c, and Dlo-RSZ20d) are localized in the chloroplasts and the remaining nine members (Dlo-SR112, Dlo-SR59, Dlo-SCL43, Dlo-RS2Z32, Dlo-RS2Z16, Dlo-RS2Z24, Dlo-SC33, Dlo-SC37 and Dlo-RS43) are localized in the nucleus and the chloroplasts. The difference in subcellular localization of the Dlo-SR proteins is suggestive of functional differences among the SR proteins. The number of predicted phosphorylation sites of longan SR protein varied substantially among the longan SR proteins, ranging from two (Dlo-RS2Z29) to 58 (Dlo-SR112) with an average of 16.

**Table 2:** Basic information of *SR* gene family members in longan.

Gene No.	Gene Name	Length (bp)	Protein length	Intron	Exon	Molecular Weight (kDa)	Isoelectric point	Instability index	Predicted Subcellular location(s)
**Dlo_036683.1**	*Dlo-RSZ20a*	-	175	-	-	19.85	11.45	99.39	Nucleus
**Dlo_037716.1**	*Dlo-RSZ20b*	-	179	-	-	20.23	11.45	97.63	Chloroplast
**Dlo_038040.1**	*Dlo-RSZ20c*	-	178	-	-	20.18	11.45	98.97	Chloroplast
**Dlo_038060.1**	*Dlo-RSZ20d*	-	179	-	-	20.23	11.45	97.63	Chloroplast
**Dlo_007311.1**	*Dlo-RS43*	2821	370	4	5	42.7	10.67	78.42	Chloroplast, Nucleus
**Dlo_000762.1**	*Dlo-SC18*	3045	155	3	4	17.9	9.63	45.76	Chloroplast
**Dlo_007965.1**	*Dlo-SC33*	3267	263	5	6	30.33	11.01	76.47	Chloroplast, Nucleus
**Dlo_020652.1**	*Dlo-SC37*	3391	320	7	8	37.28	10.75	90	Chloroplast, Nucleus
**Dlo_008139.4**	*Dlo-RS2Z29*	2662	260	6	7	28.74	9.21	46.99	Chloroplast
**Dlo_029439.1**	*Dlo-RS2Z32*	1881	286	6	7	31.67	9.57	72.3	Chloroplast, Nucleus
**Dlo_004147.1**	*Dlo-RS2Z16*	868	141	4	5	16.16	11.11	73.31	Chloroplast, Nucleus
**Dlo_010896.1**	*Dlo-RS2Z24*	2270	210	3	4	24.14	10.59	88.9	Chloroplast, Nucleus
**Dlo_009794.2**	*Dlo-SCL41*	4060	370	6	7	41.46	9.74	62.29	Chloroplast
**Dlo_021971.1**	*Dlo-SCL43*	6776	386	9	10	43.01	12.28	147.74	Chloroplast Nucleus
**Dlo_022256.1**	*Dlo-SR112*	4803	1013	4	5	112.05	10.01	74.51	Chloroplast Nucleus
**Dlo_030358.1**	*Dlo-SR30*	2227	252	3	4	29.8	10.03	68.2	Nucleus
**Dlo_021112.1**	*Dlo-SR59*	2770	523	2	3	59.19	8.18	79.27	Chloroplast Nucleus
**Dlo_032826.1**	*Dlo-SR17*	2315	154	5	6	17.23	11.84	132.3	Nucleus
**Dlo_006464.1**	*Dlo-SR33*	2342	288	9	10	33.31	10.52	105.39	Chloroplast
**Dlo_006257.1**	*Dlo-SR53*	8776	470	7	8	53.43	6.19	41.09	Nucleus
**Dlo_006259.1**	*Dlo-SR32*	3063	282	10	11	31.61	11.11	88.46	Nucleus

### 3.2 Phylogenetic and conserved motif analysis of *Dlo-SR* gene family members

**Fig 1:**
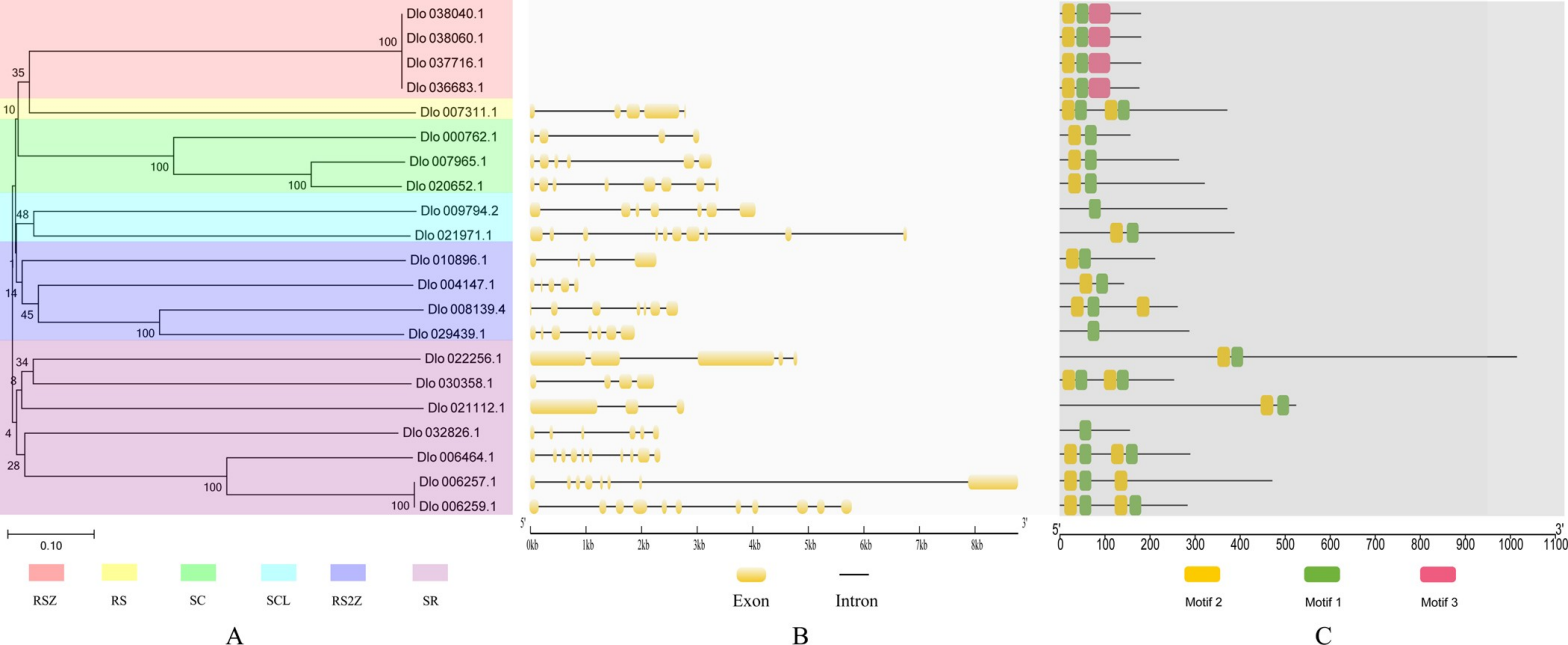
Schematic representation of the phylogenetic tree, exon-intron compositions and conserved motifs in longan SR genes. A. Neighbour-joining (NJ) phylogenetic tree for SR. B. Exon-intron organization of SR. C. Distribution of conserved motifs in SR proteins.

To analyze evolutionary relationships between the *SR* gene families of longan and *Arabidopsis*, a phylogenetic tree was constructed using the neighbor-joining method (Fig 1A). Consistent with the nomenclature for *Arabidopsis* SR proteins [7], *Dlo-SR* gene family members were named systematically according to the phylogenetic tree and the molecular weight (Table 2). The *Dlo-SR* genes consisted of six subfamilies: RSZ, RS, SC, RS2Z, SCL and SR. Among these subfamilies, RS, RS2Z and SCL are plant-specific [7]. The number of *Dlo-SR* members in each subfamily ranged from one (RS subfamily) to seven (SR subfamily). Bootstrap analysis supported a close relationship between the members of the RSZ and SC subfamilies, whereas the genetic distances indicated that the SCL subfamily members were distantly related. The members of the RS2Z and SR subfamilies were closely or distantly related, exhibiting conserve or protein-specific characteristics.

Exon and intron positions in longan *SR* genes were mapped using the GSDS server. The number of introns ranged from two to ten and the number of exons ranged from three to 11 (Fig 1B). The genes that contained the highest introns and fewest exons were *Dlo-SR32* and *Dlo-SR59*, respectively. Three conserved motifs in the Dlo-SR proteins were predicted, namely Motif 1 (RPRGFAFVEFEDRRDAEDAIRALDGKN), Motif 2 (LYVGNLSPRVTERELEDLFSKYGKVVDVD), and Motif 3 (GWRVELSHNSKGGGGRGGARGRGGGEDLKCYECPGHFARECRLRVGS) (Fig 1C). Each motif comprised 27, 29, and 49 amino acids,e respectively. All four RSZ subfamily members contained the three conserved motifs. Dlo-SR17 in the SR subfamily, Dlo-SCL41 in the SCL subfamily and Dlo-RS2Z32 in the RS2Z subfamily contained only Motif 1, and the remaining gene family members contained Motif 1 and Motif 2.

### 3.3 Predication of c*is*-acting elements in the *Dlo-SR* gene promoter

The PlantCARE database was used to analyze the 2 kb upstream sequence of the 21 *Dlo-SR* gene family members to predict the transcription start site (TSS) (Table 3). The 21 *Dlo-SR* genes all contained the core promoter elements CAAT-Box and TATA-Box. In addition to *Dlo-RS2Z16*, promoters of the other *Dlo-SR* genes contained multiple *cis*-acting elements related to growth and development, and responsive to phytohormones, stress, and light. In addition, the promoter of each *Dlo-SR* gene contained a large number of *cis*-acting elements of unknown functions, therefore the functions of *Dlo-SR* genes remain to be elucidated.

**Table 3:** Prediction of *cis*-acting elements of SR promotors in longan.

SR subfamily	SR Gene	Unknown element	Core promoter element*	Photo-response element	Development, plant hormones, stress response components
**RSZ subfamily**	***Dlo-RSZ20a***	15	2	8	7
	***Dlo-RSZ20b***	15	2	8	7
	***Dlo-RSZ20c***	15	2	8	7
	***Dlo-RSZ20d***	15	2	8	7
**RS subfamily**	***Dlo-RS43***	10	2	5	9
**SC subfamily**	***Dlo-SC18***	12	2	7	7
	***Dlo-SC33***	16	2	3	6
	***Dlo-SC37***	11	2	6	7
**RS2Z subfamily**	***Dlo-RS2Z29***	11	2	12	10
	***Dlo-RS2Z32***	14	2	7	9
	***Dlo-RS2Z16***	5	2	0	1
	***Dlo-RS2Z24***	10	2	3	5
**SCL subfamily**	***Dlo-SCL41***	13	2	10	4
	***Dlo-SCL43***	12	2	8	7
**SR subfamily**	***Dlo-SR112***	13	2	9	9
	***Dlo-SR30***	13	2	8	6
	***Dlo-SR59***	14	2	6	8
	***Dlo-SR17***	5	2	9	5
	***Dlo-SR33***	10	2	6	6
	***Dlo-SR53***	9	2	9	3
	***Dlo-SR32***	8	2	9	3

* Core promoter element: CAAT-Box, TATA-Box

### 3.4 Expression pattern of *Dlo-SR* genes in different tissues of longan

**Fig 2:**
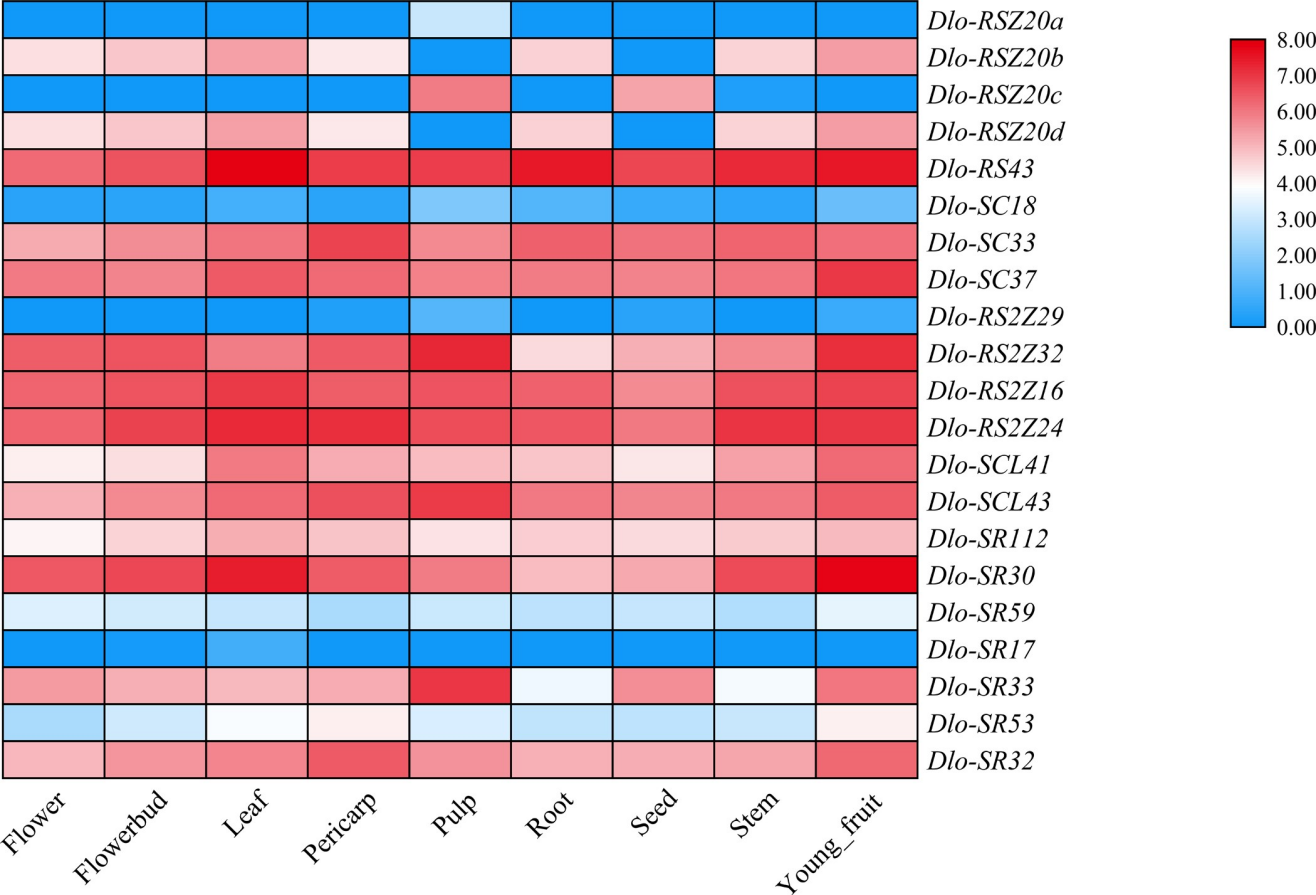
The expression pattern of *Dlo-SR* genes in different tissues of longan.

The transcript levels of *Dlo-SR* genes in the root, stem, leaf, flower, flower bud, pericarp, pulp, seed, and young fruit of longan indicated that the expression levels varied in different tissues (Fig 2). The expression levels of *Dlo-RSZ20a*, *Dlo-RSZ20b*, *Dlo-SR17*, *Dlo-SC18*, and *Dlo-RS2Z29* were low in all analyzed organs, whereas the transcript levels of *Dlo-RS43*, *Dlo-RS2Z16*, *Dlo-RS2Z24*, *Dlo-SCL43*, *Dlo-SR3*0, and *Dlo-SR32* were high in all tissues. In the same subfamily, the transcript level of *SR* genes differed, for example, in the SC subfamily, the abundance of *Dlo-SC18* transcripts was distinctly lower than that of *Dlo-SC33* and *Dlo-SC37*, and in the RS2Z family, *RS2Z16*, *RS2Z32*, and *RS2Z24* transcripts were more abundant than *RS2Z29* transcripts. Certain *Dlo-SR* genes exhibited a tissue-specific expression pattern and transcription of different members in the same family varied in the same tissue. In the pulp, the expression level of *Dlo-RSZ20a* and *Dlo-RSZ20c* were higher than those of *Dlo-RSZ20b* and *Dlo-RSZ20b* (all members of the RSZ family). In the SR family, the expression level of *Dlo-SR33* was highest in the pulp, followed by that of *Dlo-SR30*, *Dlo-SR32*, and *Dlo-SR112*.

### 3.5 Expression analysis of *Dlo-SR* genes at early stages of longan somatic embryogenesis

**Fig 3:**
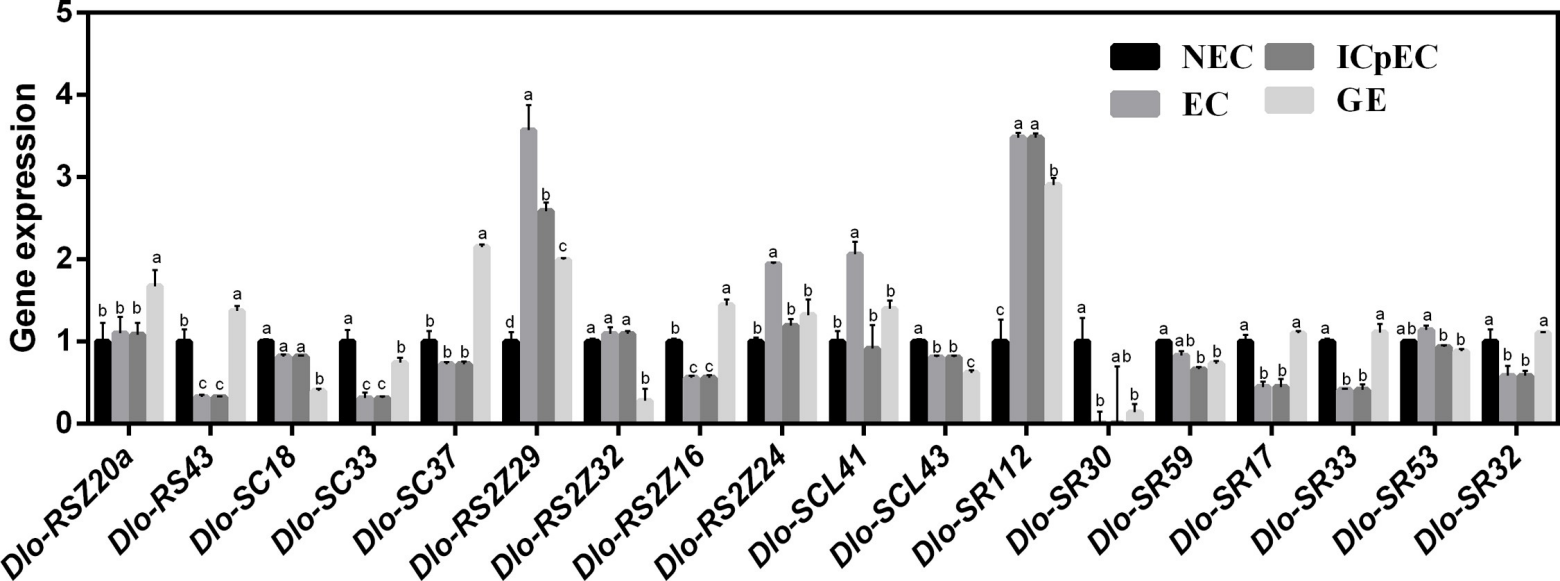
Expression analysis of *Dlo-SR* genes in the somatic embryogenesis of longan including non-embryonic callus (NEC) stage, friable-embryogenic callus (EC) stage, incomplete compact pro-embryogenic cultures (ICpEC) stage and globular embryos (GE) stage. The gene expression at NEC stage was used as control. ACTB was used as internal controls for calculating the relative expression of SR following the 2^−△△CT^ method. The data presented as average ± standard error of three replicates.

To investigate the role of *Dlo-SR* genes during longan embryogenesis, qRT-PCR analysis of 18 *Dlo-SR* genes was performed at the early stages of SE, namely NEC, EC, ICpEC and GE, with genes at the NEC stage as a control (Fig 3). Compared with that at the NEC stage, most transcripts of *Dlo-RS2Z29* and *Dlo-SR112* was detected in embryonic stages (EC, ICpEC and GE). Moreover, the specific induction of *Dlo-RS2Z24* and *Dlo-SCL41* was noted at the EC stage while the expression level of *Dlo-RSZ20a* and *Dlo-SC37* and *Dlo-RS2Z16* was highest at the GE stage. In addition, the expression level of *Dlo-RS43*, *Dlo-SC33*, *Dlo-RS2Z16*, *Dlo-SCL43*, *Dlo-SR17*, *Dlo-SR33*, and *Dlo-SR32* was reduced at the ICpEC and EC stages compared with that at the NEC stage.

### 3.6 Analysis of AS events of *Dlo-SR* genes during longan somatic embryogenesis

**Fig 4:**
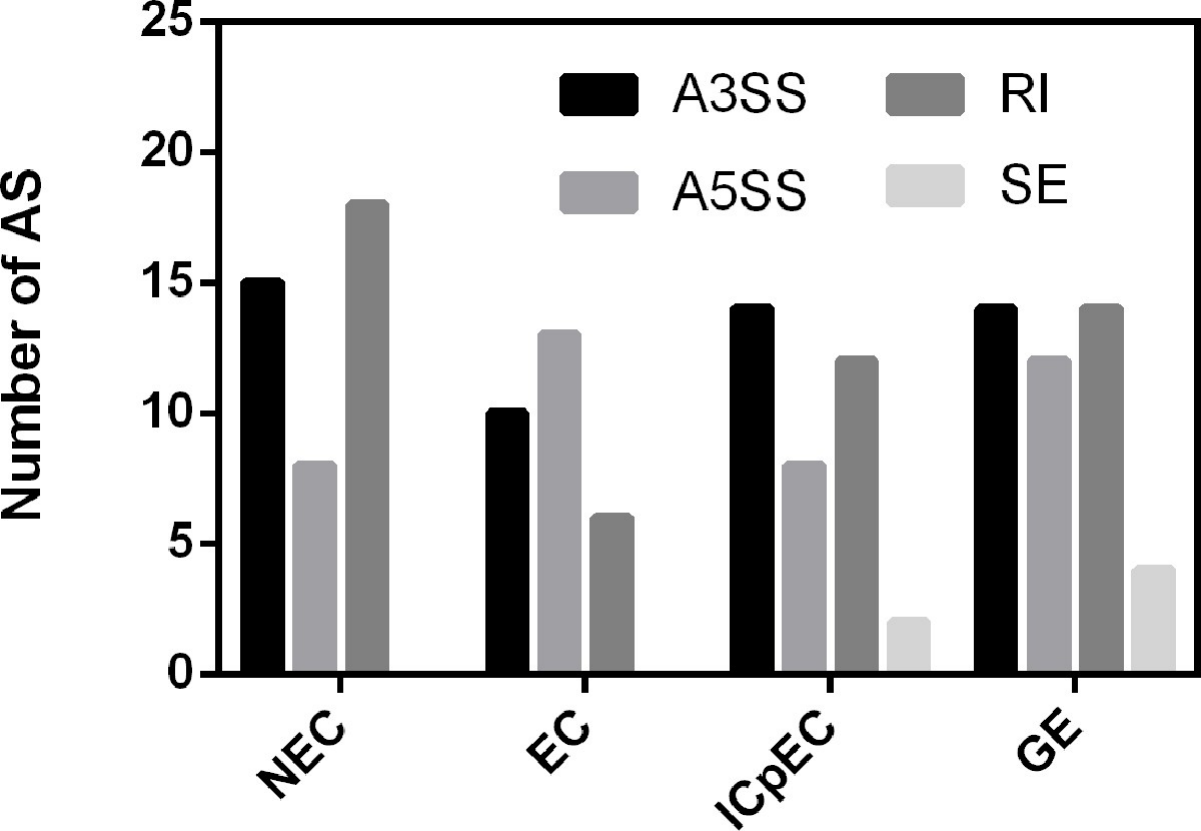
Types and number of AS events of *Dlo-SR* in somatic embryo of longan at different stages. NEC: Non-embryonic callus, EC: friable-embryogenic callus, ICpEC: incomplete compact pro-embryogenic cultures, GE: globular embryos.

Alternative splicing can be divided into seven predominant forms according to the location of splicing in the mRNA precursor (pre-mRNA) sequence: exon skipping (ES), retention intron (RI), alternative 5'splice site (A5SS), alternative 3'splice site (A3SS), alternative first exon, alternative last exon and mutually exclusive exon. The first four AS forms are the predominant types observed in eukaryotes. The AS events for *Dlo-SR* gene family members were analyzed at four stages including NEC, EC, ICpEC, and GE (Fig 4 and Tables 4 and S2). Forty-one AS events (15 A3SS, eight A5SS, and 18 RI) occurred in pre-mRNAs of 14 *Dlo-SR* genes during the NEC stage, 29 AS events (10 A3SS, 13 A5SS, and six RI) in pre-mRNAs of 14 *Dlo-SR* genes during the EC stage, 35 AS (13 A3SS, 8 A5SS, 12 RI, and two ES) in pre-mRNAs of 13 *Dlo-SR* genes during the ICpEC stage, 44 AS (14 A3SS, 12 A5SS, 14 RI, and four ES) in pre-mRNAs of 16 *Dlo-SR* genes during the GE stage. The main types of AS of *Dlo-SR* genes varied at the different stages (Fig 4). Alternative splicing of *Dlo-SR* pre-mRNAs at the NEC stage was dominated by RI events, accounting for 43.90% of the total. The dominant type of AS of *Dlo-SR* pre-mRNAs during the EC stage was A5SS events, accounting for 44.83% of the total. No ES events of *Dlo-SR* pre-mRNAs were detected at the NEC and EC stages. The predominant type of AS of *Dlo-SR* pre-mRNAs in the ICpEC stage was A3SS events (37.14%). The A3SS and RI types were the main AS events of *Dlo-SR* pre-mRNAs at the GE stage, followed by A5SS and ES events. The incidence of RI events of *Dlo-SR* pre-mRNAs plummeted by two-thirds when the cells progressed from the NEC stage to the EC stage, suggesting that RI events of *Dlo-SR* pre-mRNAs may play an important regulatory role during SE.

**Table 4:** Types and numbers of AS events in the longan *SR* gene family members during NEC, EC, ICpEC and GE.

*SR* genes	A3SS				A5SS				RI				ES			
	NEC	EC	ICpEC	GE	NEC	EC	ICpEC	GE	NEC	EC	ICpEC	GE	NEC	EC	**ICpEC**	**GE**
***Dlo-RS43***	2	2	4	1	1	2	1	1	1			1				
***Dlo-SC18***	2			1												
***Dlo-SC33***	2	1	2	2			1	1								1
***Dlo-SC37***						1									1	1
***Dlo-RS2Z29***		1	1	1		1	1	4	2	1	1				1	1
***Dlo-RS2Z32***	3	1	2	2	4	4	4	3	2	2	1	1				
***Dlo-RS2Z16***			1						1	1	1	1				
***Dlo-RS2Z24***	1	1	1	2					2		2	2				
***Dlo-SCL41***	1			1		1	1		1		1	2				1
***Dlo-SCL43***	1	2	1	1				1		1	1					
***Dlo-SR112***					1	1			3		1	1				
***Dlo-SR30***	2	1	1	1	1	1		1	1		1	1				
***Dlo-SR59***	1	1		1												
***Dlo-SR17***										1	2	3				
***Dlo-SR33***				1	1	2		1	4							
***Dlo-SR32***									1		1	2				

### 3.7 Protein–protein interaction analysis of Dlo-SR in longan

**Fig 5:**
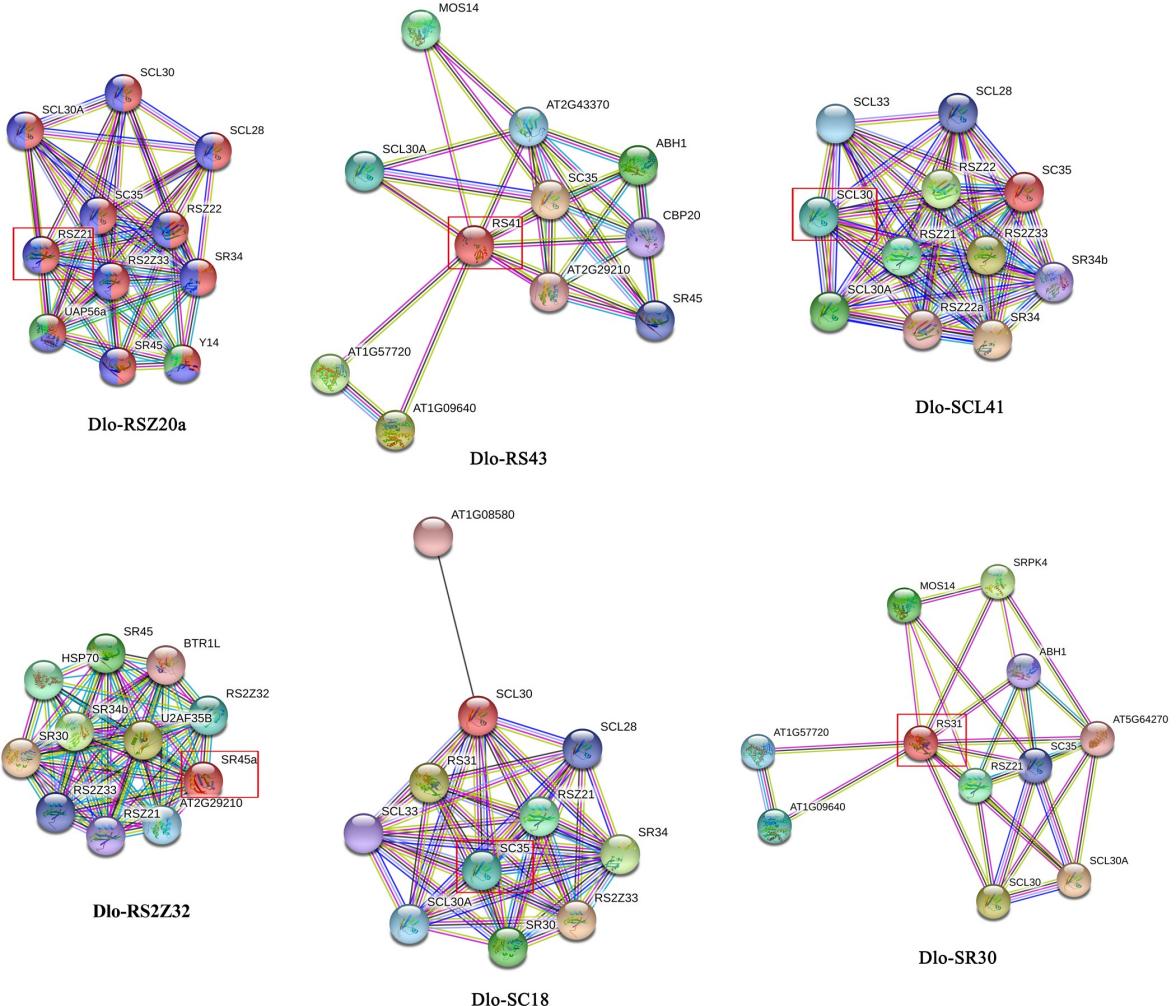
Interaction network of six selected SR proteins. STRING (https://string-db.org/) was used to construct the functional protein association networks of SR on the basis of *Arabidopsis* orthologs. Medium confidence was setting to 0.400 and max number of interactors was no more than 10.

To explore the potential functions of Dlo-SR proteins, six genes (*Dlo-RSZ20a*, *Dlo-RS43*, *Dlo-SC18*, *Dlo-RS2Z32*, *Dlo-SCL41*, and *Dlo-SR30*) from different subfamilies were selected to construct a protein–protein interaction network using STRING 11 software based on an *Arabidopsis* association model (Fig 5). The Dlo-RSZ20a protein showed high homology with At-RSZ21, and the protein interaction network consisted of 11 nodes and 50 edges. The biological processes involved RNA splicing, mRNA processing, mRNA splicing via a spliceosome and mRNA transport. Dlo-RS43 showed high homology with At-RS41, and the protein interaction network consisted of 11 nodes and 31 edges. The biological processes involved mRNA splicing via a spliceosome, primary miRNA processing, gene expression, cellular response to dsRNA and cellular nitrogen compound metabolic process. Dlo-SC18 showed high homology with At-SC35, and the protein interaction network consisted of 11 nodes and 54 edges. The biological processes involved RNA splicing, mRNA processing and mRNA splicing via a spliceosome. Dlo-RS2Z32 showed high homology with At-SR45a, and the protein interaction network consisted of 11 nodes and 55 edges. The biological processes involved RNA splicing, mRNA processing, mRNA splicing via a spliceosome, gene expression and RNA metabolic process. Dlo-SCL41 showed high homology with At-SCL30, and the protein interaction network consisted of 11 nodes and 46 edges. The biological processes involved RNA splicing, mRNA processing and mRNA splicing via a spliceosome. Dlo-SR30 showed high homology with At-RS31, and the protein interaction network consisted of 11 nodes and 29 edges. The biological processes involved mRNA splicing via a spliceosome, gene expression, cellular nitrogen compound metabolic process, translational elongation and glutathione metabolic process.

## 4 Discussion

Alternative splicing events occur extensively in genes involved in expression regulation of eukaryotes, such as signaling, programmed cell death and other related genes [34–37]. Alternative splicing also affects the efficiency, stability and localization of *cis*-acting elements in the transport and translation of mRNA [37]. In the present study, 21 members of the *Dlo-SR* gene family belonging to six subfamilies were identified in longan based on the results of a neighbor-joining analysis with *At-SR* genes of *Arabidopsis*. The *SR* gene families of longan and *Arabidopsis* are relatively conserved in evolution. The AS events of *Dlo-SR* pre-mRNAs at the NEC, EC, ICpEC, and GE stages in longan were analyzed in this study, which confirmed the involvement of *Dlo-SR* and AS in longan SE.

In plants, the study of the functions of *SR* genes is in its infancy. The mechanism of SR function in SE has not been reported in plants, but has been studied during embryogenesis in *Caenorhabditis elegans* and mouse. Longman et al. observed that RNA interference with *Ce-SF2/ASF* may be lethal in the late embryonic stage [8]. Jumaa et al. attempted to raise *srp20-*deficient mice using gene knockout technology, but failed to obtain viable offspring [9]. In the present study, we observed that during longan SE, except for *Dlo-RSZ20a* and *Dlo-SR53*, the remaining 16 *Dlo-SR* genes showed varied expression levels in the ICpEC, EC, and GE stages. It is worth noting that the expression level of *Dlo-RS2Z29* and *Dlo-SR112* were significantly increased in the embryonic cells (EC, ICpEC, and GE) compared with those in NEC, though small amounts of *Dlo-RS2Z29* transcripts were detected in different tissues of longan, which indicated it may function in an embryo-specific manner. Transcripts of the genes *Dlo-SR43*, *Dlo-SC33*, *Dlo-RS2Z16*, *Dlo-SCL43*, *Dlo-SR33*, and *Dlo-SR32* were detected in all tissues and showed varying degrees of reduction at the stages of ICpEC and EC while the transcripts of *Dlo-RSZ20a*, *Dlo-SC37*, and *Dlo-RS2Z16* were highly detected at the GE stage. Differential expression pattern of *Dlo-SR* genes during SE suggested that their involvement in the regulation of longan embryogenesis.

As shown in the promoter cis-elements analysis, the majority of SR genes contain abundant photo-responsive elements. Under blue and white light conditions, the tissue-highly expressed *Dlo-RS43* and *Dlo-RS2Z24* showed no differential response compared with the control (S1 Fig). The high abundance of *Dlo-SR33*, *Dlo-SR112*, *Dlo-SR17*, and *Dlo-RS2Z32*, as well as decreased amounts of *Dlo-RSZ20d*, *Dlo-SC33*, *Dlo-SC37*, and *Dlo-SC43* at EC in response to exposure to blue light compared with those of the control (S1 Fig). The response of SR proteins to light may also associated with their localization in chloroplasts, as chloroplasts are an important plant-specific organelle and critical for photosynthesis.

Whether *SR* genes in plant play different roles to those in mammals still needs verification. The present results provided information to support their distinction. In the traditional definition of SR protein, the serine/arginine-rich RS domain is located at the C-terminus of the SR protein polypeptide chain. However, sequence analysis revealed that the RS domain of *Dlo-RS2Z16* (Dlo_004147.1) is located at the N-terminus of the polypeptide chain. It is generally considered that the majority of SR proteins are stored in the nucleus speckles in a stable state, and that some are shuttled between the nucleus and the cytoplasm. The longan SR proteins predicted to be localized in both the nucleus and chloroplasts may belong to the nucleus-cytoplasm shuttle proteins. In addition to effect of AS of pre-mRNAs, the nucleus–cytoplasm shuttle proteins also play an important role in mRNA transport, translation regulation, and mRNA stabilization and localization [38]. The serine residue of the RS domain is the substrate for a variety of protein kinases and its phosphorylation state is closely related to the splicing which is regulated by SR proteins and to the localization of the SR protein in the cell [39–41].

## 5 Conclusion

In conclusion, a total of 21 *Dlo-SR* members were identified in longan and the expression of these *Dlo-SRs* varied in different tissues of longan. During the development of longan SE, the high expression levels of *Dlo-RS2Z29* and *Dlo-SR112* were noted in the embryogenic cells (EC, ICpEC, and GE) compared with those at the NEC stage. Moreover, the transcripts of *Dlo-RSZ20a*, *Dlo-RS43*, *Dlo-SC37*, and *Dlo-RS2Z16* were increased especially at the GE stage compared with those at other embryonic stages. In addition, AS events of *Dlo-SR* pre-mRNAs were also observed during SE with the least AS occurred in the cells at the EC stage and gradual increasement in the later transition to GE stage, indicating the involvement of SR’s AS during longan SE.

## Supporting information

S1 Fig Expression of *Dlo-SRs* in longan cells at EC stage exposed to different light conditions. Longan cells at EC stages were subjected to white and blue light conditions with dark condition as a control.
(TIF)

S1 Table Sequences of *Dlo-SR*’s CDS. (XLSX)

S2 Table The alternative splicing sites of AS events occurring in NEC and early stages during longan somatic embryogenesis. (XLSX)
